# Supplementary material for: Evolutionary rescue of resistant mutants is governed by a balance between radial expansion and selection in compact populations
Source: Nat Commun. 2022 Dec 23;13:7916. doi: 10.1038/s41467-022-35484-y (PMC9789051; doi:10.1038/s41467-022-35484-y)
Supplement: Supplementary file 1 — Supplementary Information [file 41467_2022_35484_MOESM1_ESM.pdf]

Supplementary Information for

Evolutionary rescue of resistant mutants is governed by a balance between radial expansion and selection in compact populations

Serhii Aif[1,2], Nico Appold[1,2], Lucas Kampman[3,4], Oskar Hallatschek[3,4,5,*], and Jona Kayser[1,2,*]

[1]Max Planck Institute for the Science of Light & Max-Planck-Zentrum für Physik und Medizin, 91058 Erlangen, Germany
[2]Department of Physics, Friedrich-Alexander-University Erlangen-Nürnberg, 91054 Erlangen, Germany
[3]Department of Physics, University of California, Berkeley, CA 94720, USA
[4]Department of Integrative Biology, University of California, Berkeley, CA 94720, USA
[5]Peter Debye Institute for Soft Matter Physics, Leipzig University, 04103 Leipzig, Germany
[*]E-mail: jona.kayser@mpl.mpg.de or ohallats@berkeley.edu

1 Explanation of equilibrium width

Setting Eq. 4 to zero, we can solve for the radius dependent equilibrium selection coefficient

$$s_{\text{eq}}(w|r) = \sqrt{1 + \left(\frac{w}{2r}\right)^2} - 1 \tag{1}$$

(Fig. 4). A stable equilibrium at $w = w_{\text{eq}}(r)$ will only emerge if the clone is subject to a width-dependent effective selection coefficient s_{eff} in the following way: Clones fluctuating to $w > w_{\text{eq}}(r)$ need to be subjected to selection stronger than inflation and therefore reduced in size while, conversely, for clones of a size $w < w_{\text{eq}}(r)$ inflation dominates, increasing their width. Note that no constant selection coefficient can result in a stable equilibrium. It can only happen for width dependent selection or inflation.

2 Efficacy

As there is some variation in colony growth from colony to colony, survival probabilities of clones from different conditions are not necessarily measured at the same radii. It creates difficulty in efficacy calculation, as it is hard to compare two samples with different colony sizes. Though variation in colony sizes is not very big, as they are imaged with the same frequency and the majority of cells are yMG10 in all of the samples. To increase the validity of comparison of with-rescue and no-rescue experiments, we interpolate the values of survival probabilities, to compare the values at the same radii. Efficacy is then calculated as $\mathcal{E} = 1 - \frac{P_{\text{surv}}^{\text{no-escape}}}{P_{\text{surv}}^{\text{with-escape}}}$, linearly interpolating probabilities between measured points and comparing probability values at the same colony radius. Errors are calculated using Gaussian error propagation. Survival probability error consists of clone number uncertainty ($\sqrt{n}$, considering Poisson distribution) and uncertainty of colony radius. Later is taken into account by $\Delta P_R = \frac{\partial P}{\partial R}\Delta R$, where partial derivative is estimated numerically using piece-wise linear interpolation of data points.

$$\Delta P(R) = \sqrt{\Delta P^2 + \left(\frac{\partial P}{\partial R}\right)^2 \Delta R^2}$$

3 Supplementary tables

Name	Type	Colour	Resistance	Genotype
yJK26	uncomp.*	red	HYG	hoΔ::PSCW11-cre-EBD78-hygMX SUC2::P_ENO2-loxP-ymCherry-kanMX-loxP-ymCerulean-ubq-cyh2r
yJK26c	comp.*	cyan	HYG CHX	hoΔ::PSCW11-cre-EBD78-hygMX SUC2::P_ENO2-loxP-ymCerulean-ubq-cyh2r
yMG10c	"wild-type"	yellow	CHX	hoΔ::PSCW11-cre-EBD78-natMX SUC2::P_ENO2-loxP-ymCitrine-ubq-cyh2r
yJK20	ancestor	-	-	hoΔ::PSCW11-cre-EBD78-natMX SUC2::P_ENO2-loxP-ymCherry-kanMX-loxP-ymCerulean-ubq-cyh2r
yJK19	ancestor	-	-	hoΔ::PSCW11-cre-EBD78-natMX
yMG10	ancestor	-	-	hoΔ::PSCW11-cre-EBD78-natMX SUC2::P_ENO2-loxP-ymCherry-kanMX-loxP-ymCitrine-ubq-cyh2r

Supplementary Table 1: *S.cerevisiae* strains used in this work. * - uncompensated and compensated clone types.

Name	Units	Value
Fitness cost	None	0.013
Selection type	-	dynamic
Mutation rate	1/μm	0.0001
Effective selection type	-	$s_0 \cdot (1 - e^{\frac{w}{w_c}})$
Critical width	μm	280
Diffusion coefficient	μm	0.23
Cell size	μm	5
Δ_r	μm	1
Initial radius	μm	1827
Initial width distribution	-	normal
Mean initial width	μm	20
Initial width s.d.	μm	5
Number of steps	μm	7000

Supplementary Table 2: Random walk simulation parameters.

Name	Units	Value
Domain size	μm^2	6000x6000
Cell radius	μm	6
Initial colony size	μm	600
Nutrients diffusion coefficient	μm^2/min	9000000.0
Boundary conditions	-	Dirichlet, circle
Cell cycle average time (resistant)	min	1140
Cell cycle average time (wild type)	min	900

Supplementary Table 3: Parameters used in the agent based model. For more details source code is available in the repository specified in the Code availability section.

4 Supplementary Figures

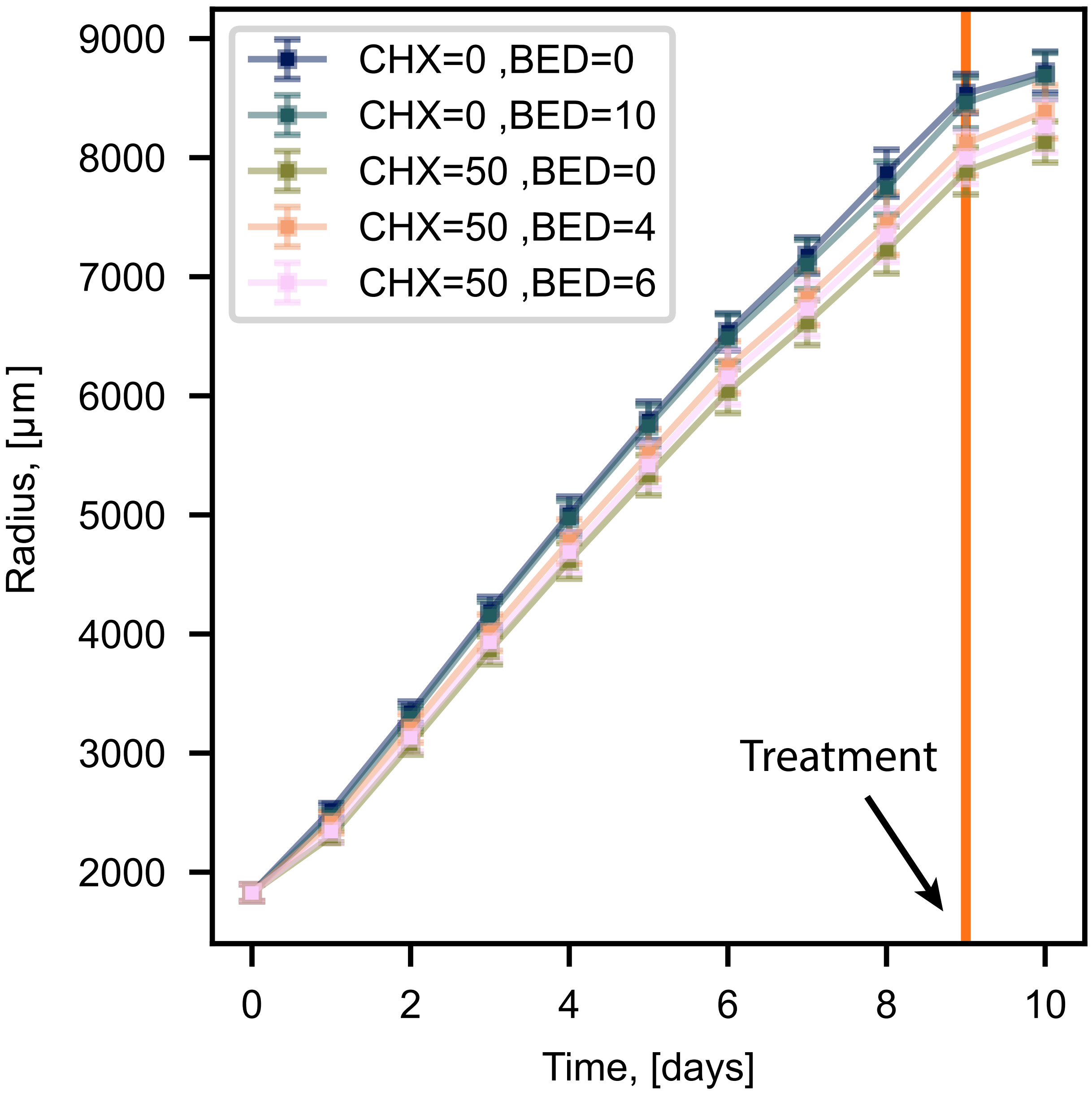

Supplementary Figure 1: **Colony growth dynamics.** Average colony radius (mean ± SD) for different days and experimental conditions. CHX and BED are concentrations of cycloheximide and β-estradiol in nM. Orange line represents treatment time point. $n = 6, 5, 18, 18, 17$ for conditions in the legend from top to bottom.

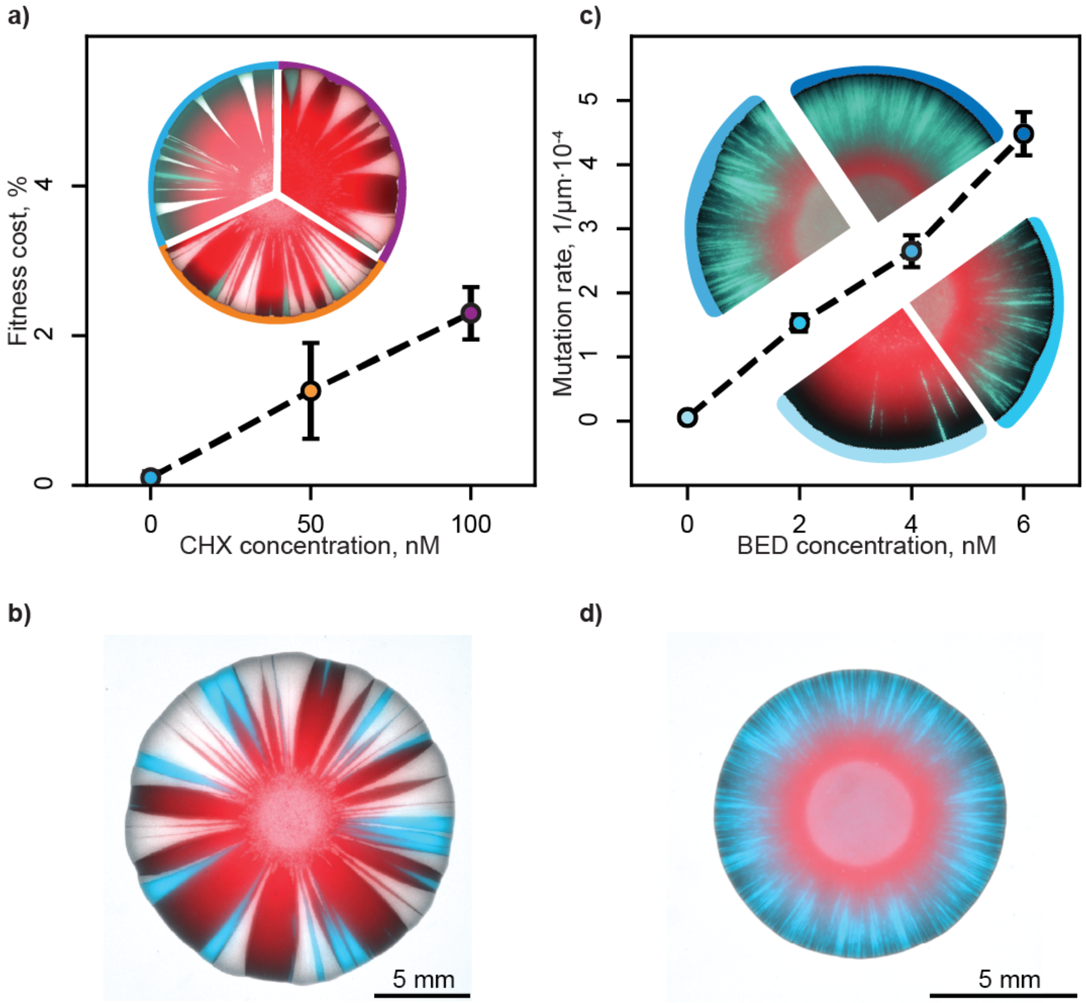

Supplementary Figure 2: **Measurements of fitness cost and mutation rate.** **a)** Fitness difference of "uncompensated" clone to the "wild-type" at different concentrations of cycloheximide (mean ± SD). $n = 7$, 9 and 5 technical replicates for cycloheximide concentrations 0, 50 and 100 respectively. Inset, parts of colonies representing cone development in experiments with respective (color coded) cycloheximide concentrations. **b)** One of the colony images from the fitness measurement experiment. Fitness cost $s = 0.013 \pm 0.006$, no β-estradiol ($\mu = 5.65 \pm 3.46 \times 10^{-6}\ \mu\text{m}^{-1}$). 5 % of wild type inoculated in 95 % of resistant mutant. Colony grown for 8 days. **c)** Mutation rates per cell per micron of radial colony growth for different concentrations of β-estradiol (mean ± SD). $n = 6$ independent colonies for each condition. Inset, parts of colonies from the respective experiments (color coded). **d)** Colony from the experiment of switching rate measurement, mutation rate - $\mu = 2.65 \pm 0.25 \times 10^{-4}\ \mu\text{m}^{-1}$. Colony grown for 4 days.

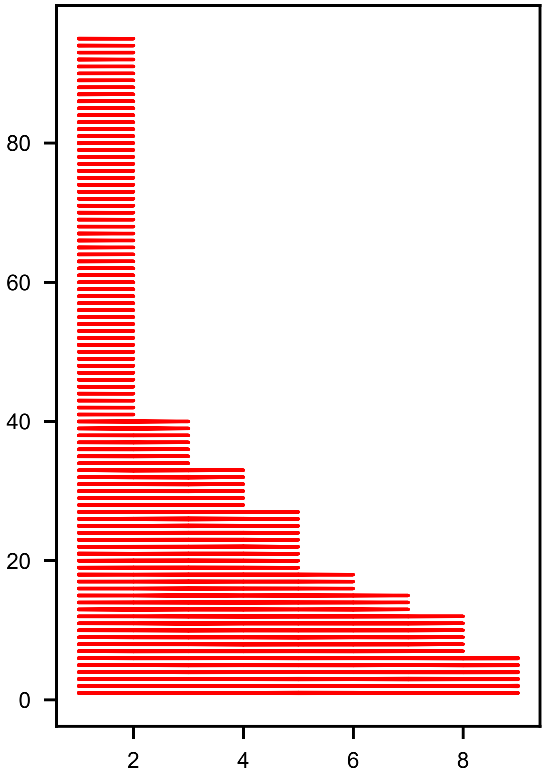

Supplementary Figure 3: **Trajectories of clones from no rescue control.** Trajectories of clones from the experiment with red clones fitness cost $s = 0.013 \pm 0.006$ and mutation rate $\mu = 5.65 \pm 3.46 \times 10^{-6} \frac{1}{\mu\text{m}}$.

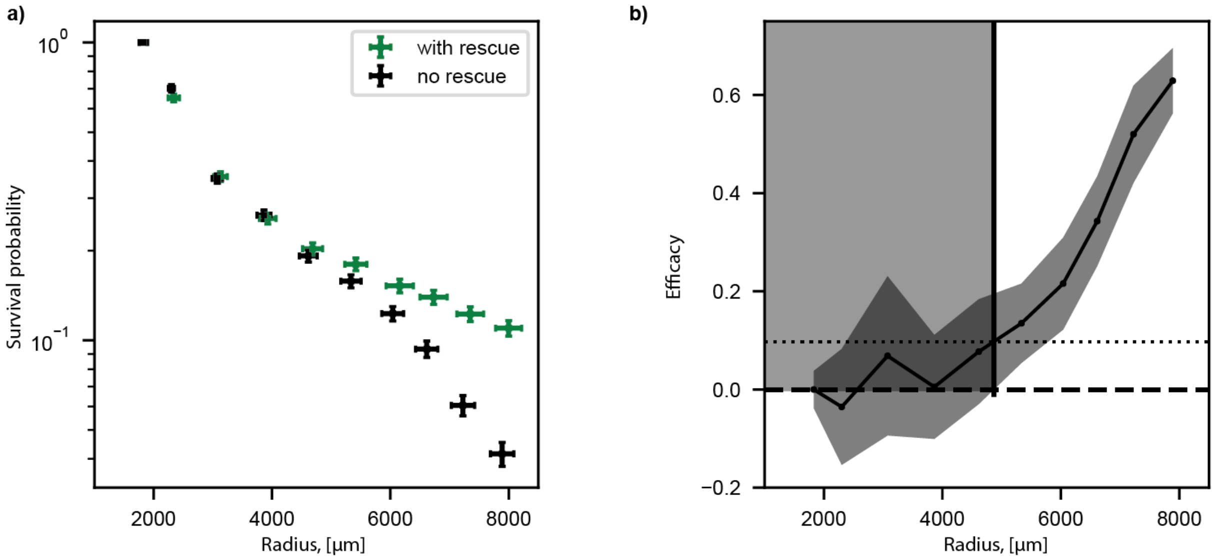

Supplementary Figure 4: **Analysis of experiment with a different mutation rate.** **a** Survival probability for "very high" (Fig. 3) mutation rate, $\mu = 4.48 \pm 0.34 \times 10^{-4} \frac{1}{\mu\text{m}}$. Error bars indicate Poisson distribution SD (vertical axis) and SD of the mean (horizontal axis), $n = 17$ independent colonies. **b** Efficacy of compensatory mutations. Shaded area indicates propagated SDs.

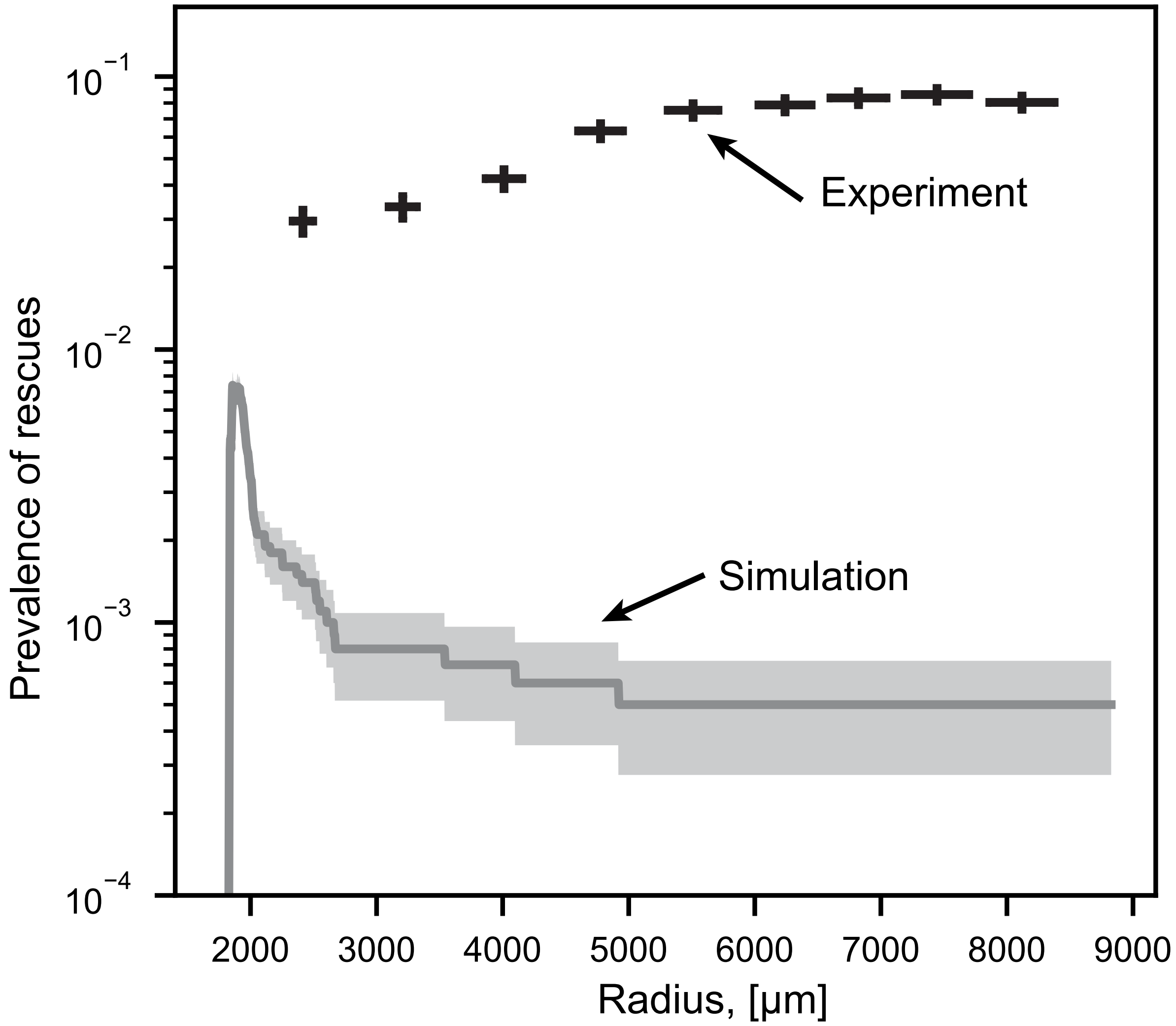

Supplementary Figure 5: **Prevalence of rescues.** Prevalence of compensatory mutations defined as the ratio of compensated clones at a given radius to initial total number of clones. Solid line - random walk model, individual points - experimental data. $n = 18$ independent colonies. Error bars/shaded area indicate Poisson distribution SD (vertical axis) and SD of the mean (horizontal axis).

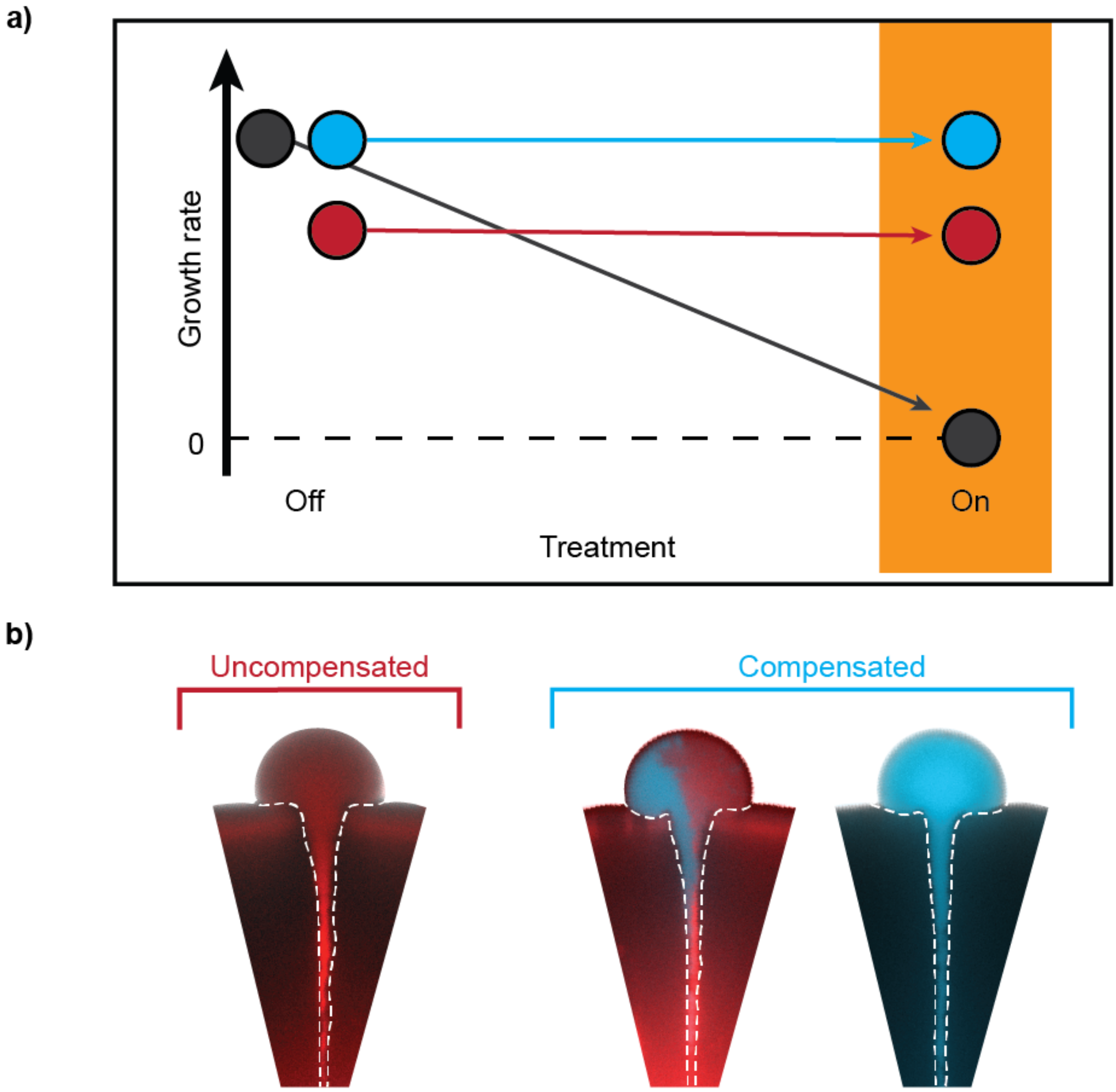

Supplementary Figure 6: **Treatment mimicry in the experiment.** **a)** Schematics of clone growth rates before and after treatment application. **b)** Treatment failure event classification into compensated and uncompensated.

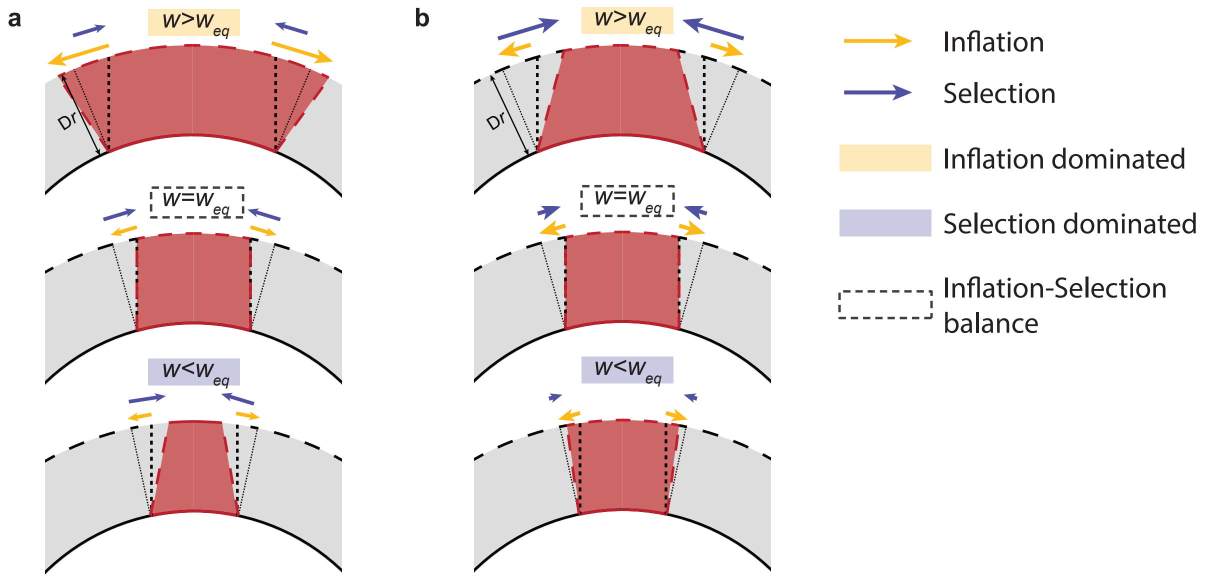

Supplementary Figure 7: **Inflation-selection-balance schematics.** **a)** Unstable and**b)** stable equilibrium clone width dynamics. Arrows represent directions, in which inflation or selection are acting on the clone.

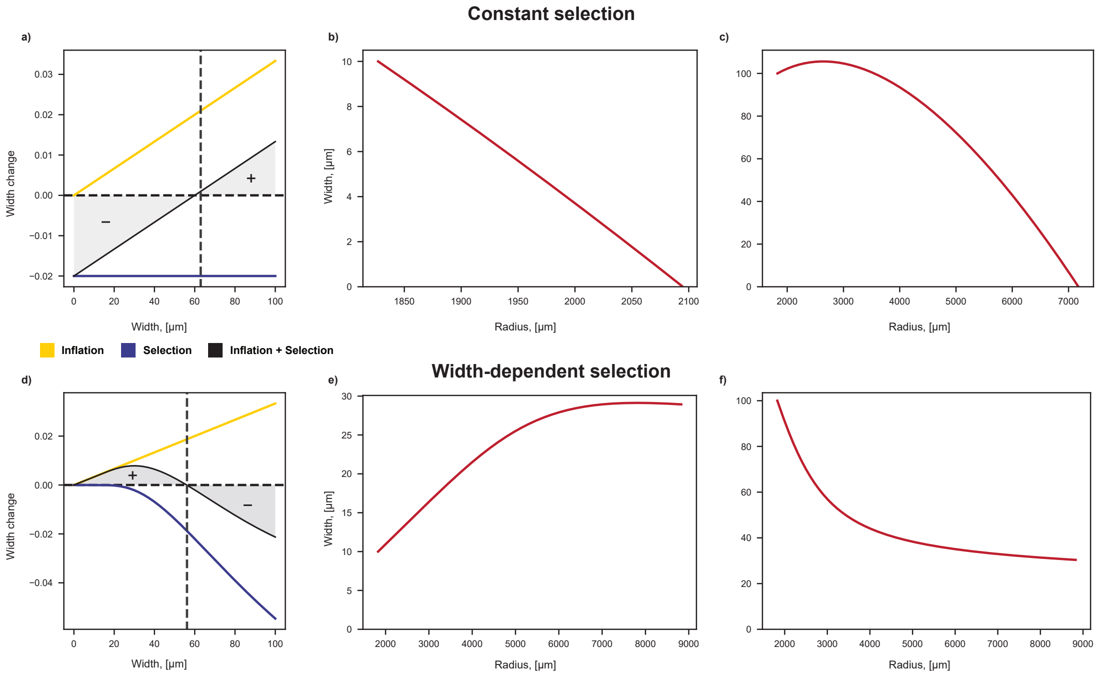

Supplementary Figure 8: **Random walk: constant and width-dependent selection.** Clone width change as the function of its width. Yellow line - pure inflation contribution, blue - contribution of selection, black - sum of the two. Plus indicates the region, where clone width increases, minus - decreases. Zero of Inflation + Selection is an equilibrium of two forces. **a)** Constant selection ($s_0 = 2 \cdot 10^{-4}$) leads to unstable equilibrium width. **b),c)** Represent clone width dynamics in time, while radius grows and slope of inflation gets smaller, starting at different initial clone width. Noise is turned off. **d** Width-dependent selection of the form of function $s_{\text{eff}} = s_0 \cdot \frac{2}{1+e^{\frac{w_c}{w}}}$. $s_0 = 1.3 \cdot 10^{-2}$, $w_c = 280\,\mu\text{m}$. **e),f)** Clone width dynamics starting with different initial width.

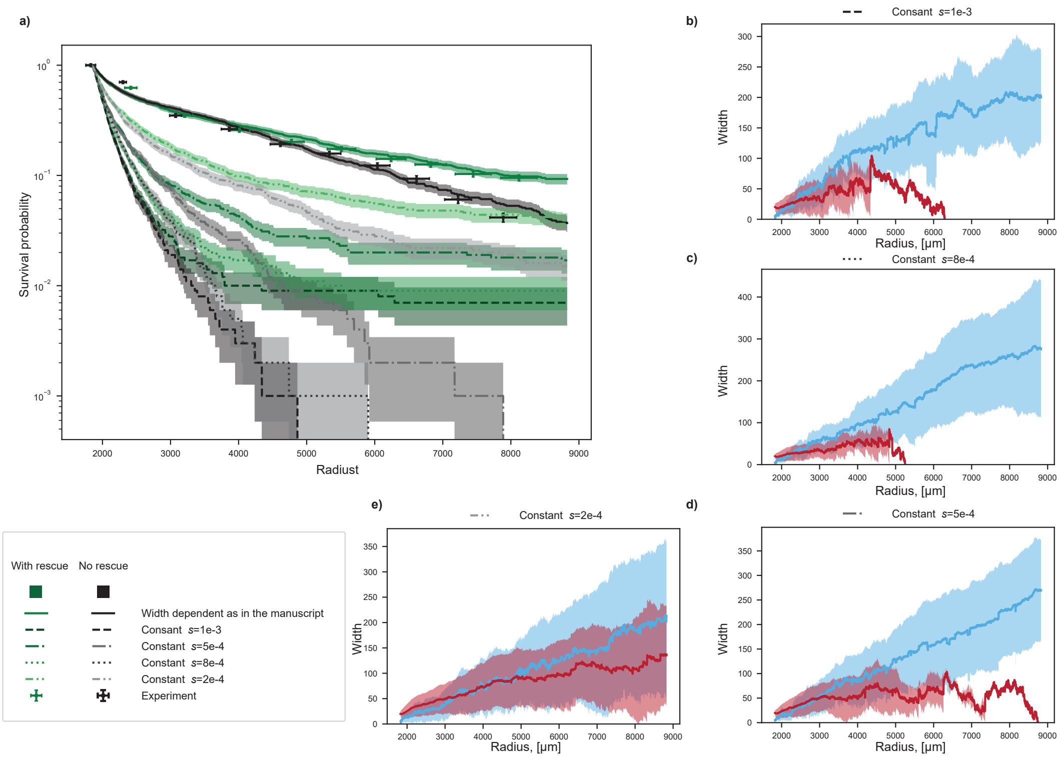

Supplementary Figure 9: **Comparison to null model with different width-independent selection coefficients**. **a)** Survival probabilities: experiment - dots (data identical to Figure 2 in the main text, $n = 18$ independent colonies), random walk with parameters used in the main manuscript - solid lines, and random walk with constant, but reduced selection - different line styles. Shaded area represents Poisson distribution SD **b-e)** Average width dynamics for different scenarios of reduced constant selection for uncompensated (red) and compensated (blue) clones. Shaded areas indicate interquartile ranges.

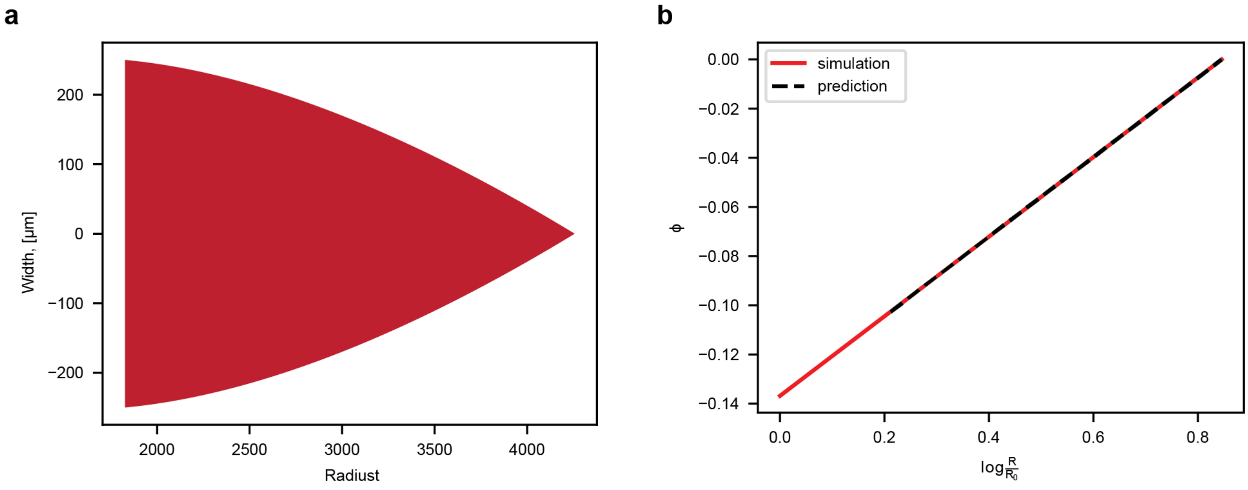

Supplementary Figure 10: **Example of clone width dynamics for random walk simulation with constant selection.** **a)** Illustration of a large clone quickly shrinking to extinction in random walk model with experimental value of constant selection. **b)** Comparison of the null model (without noise) clone angle (simulation) to the theory from the reference [1] (log spiral fit). Selection coefficient $s_0 = 0.013$.

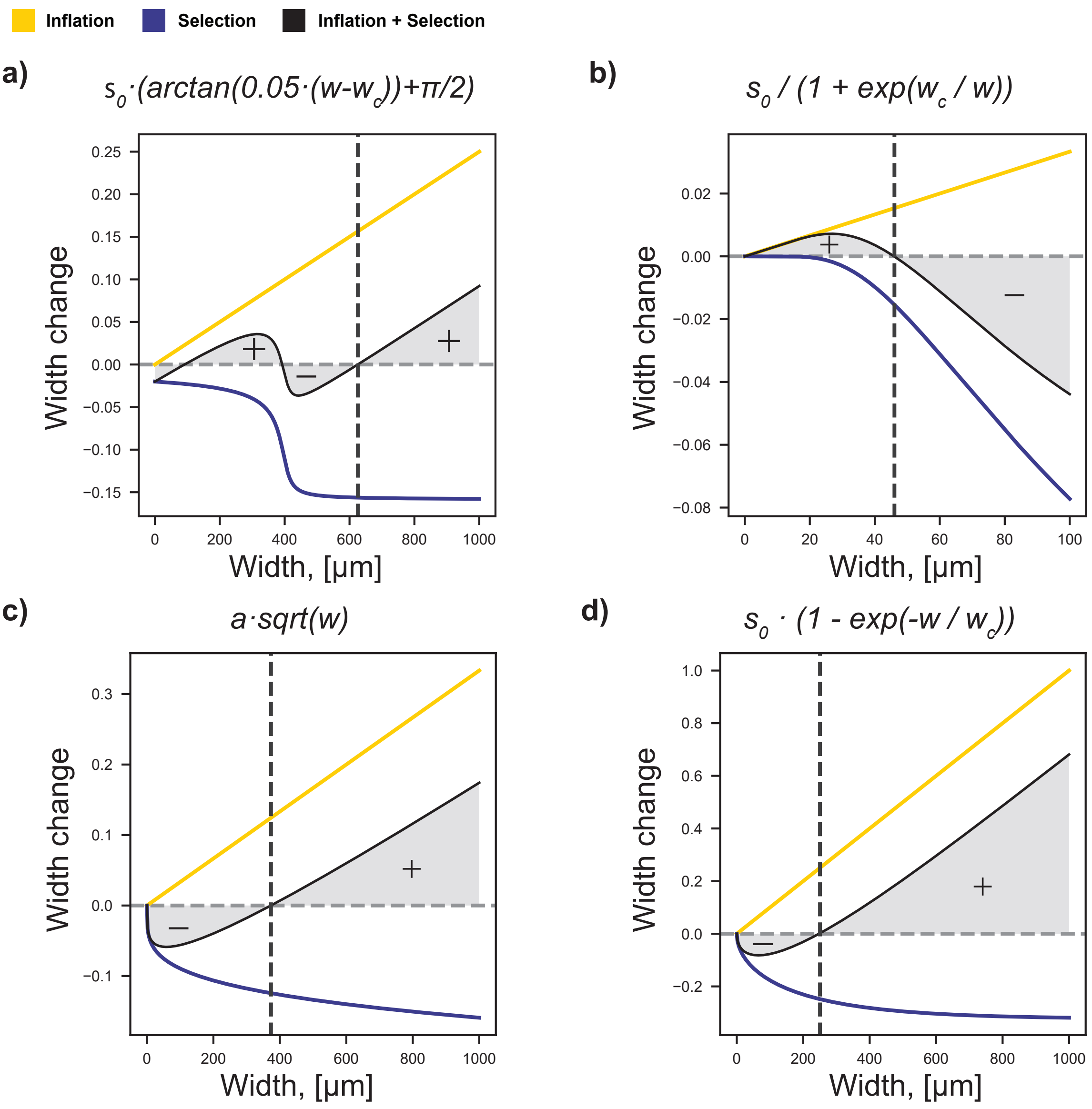

Supplementary Figure 11: **Comparison of different effective selection shapes.** Clone width change as the function of clone's size for different effective selection shape. Yellow line - pure inflation contribution, blue - pure selection contribution, black - total width change. Plus and minus indicate areas of growing and shrinking clones. **a-d)** different forms of effective selection.

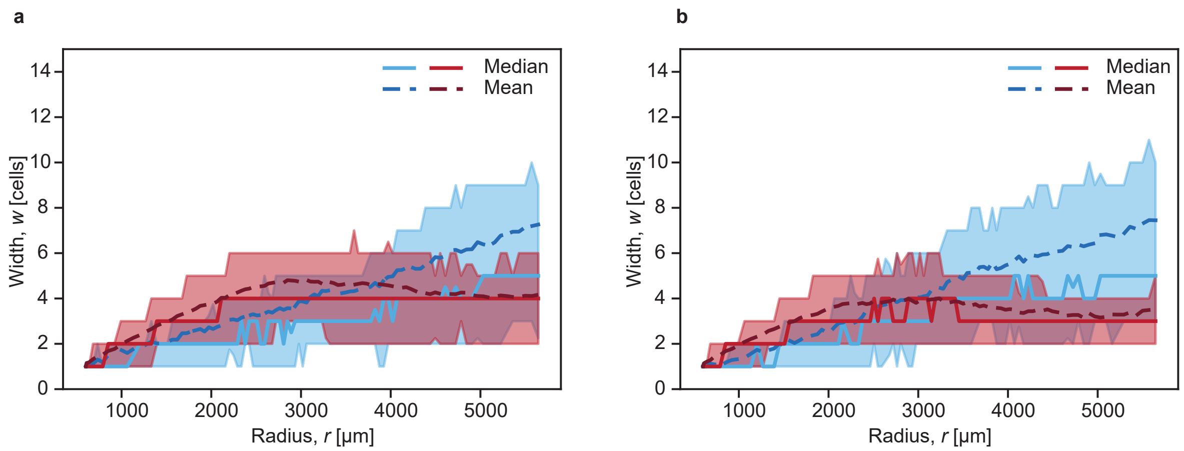

Supplementary Figure 12: **Average and median width dynamics**. Median and mean width development of blue and red clones in agent-based simulations. **a** Fitness cost of 0.11 , **b** fitness cost of 0.16. Shaded areas indicate interquartile ranges.

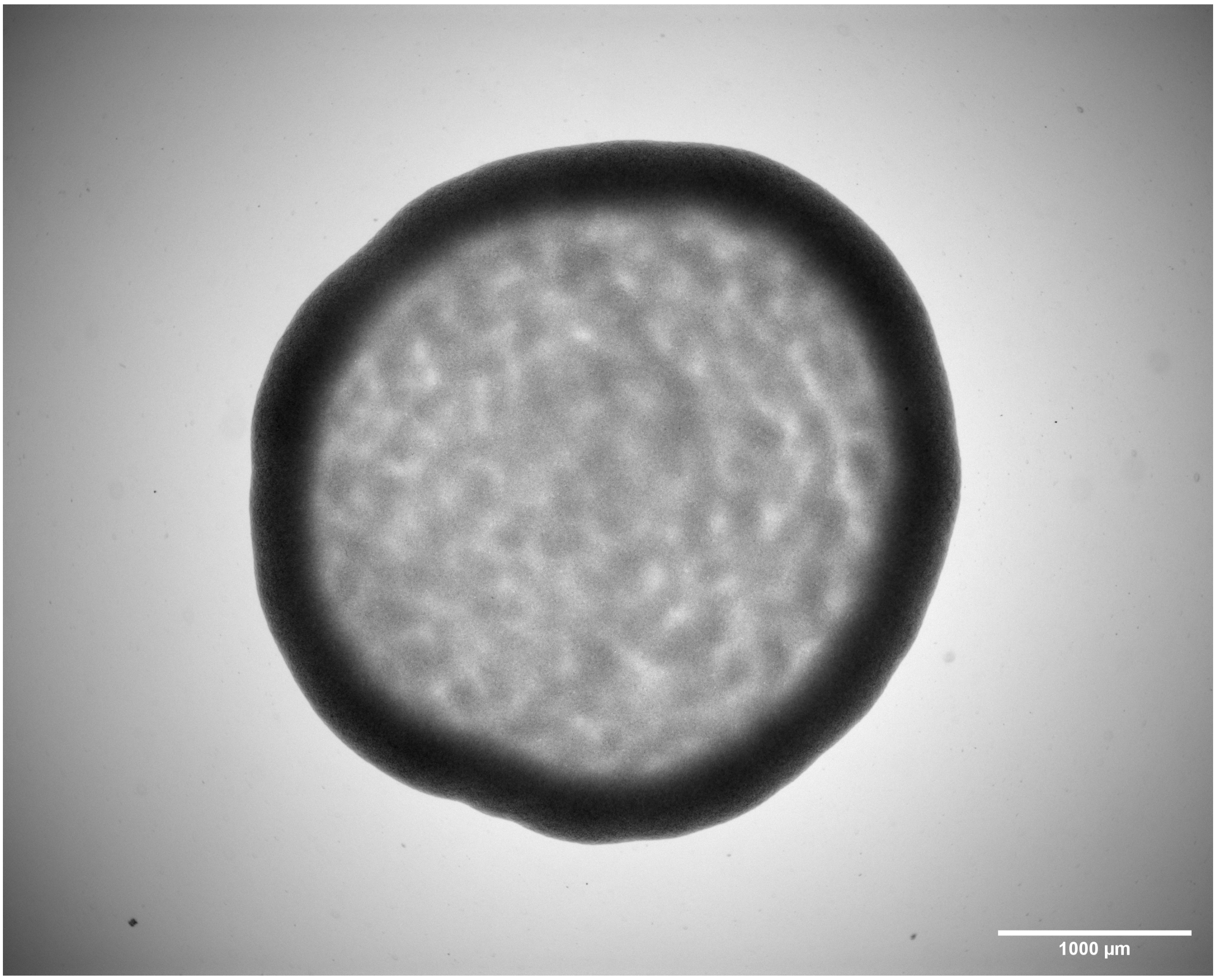

Supplementary Figure 13: **Colony after inoculation.** Bright-field image of a colony ∼ 3 hours after innoculation. High density of the cells in the periphery of the colony is observed due to the "coffee ring effect". Each colony that was used for the analysis in the main results section (n=48) was imaged 1-3 hours after inoculation and looked similar.

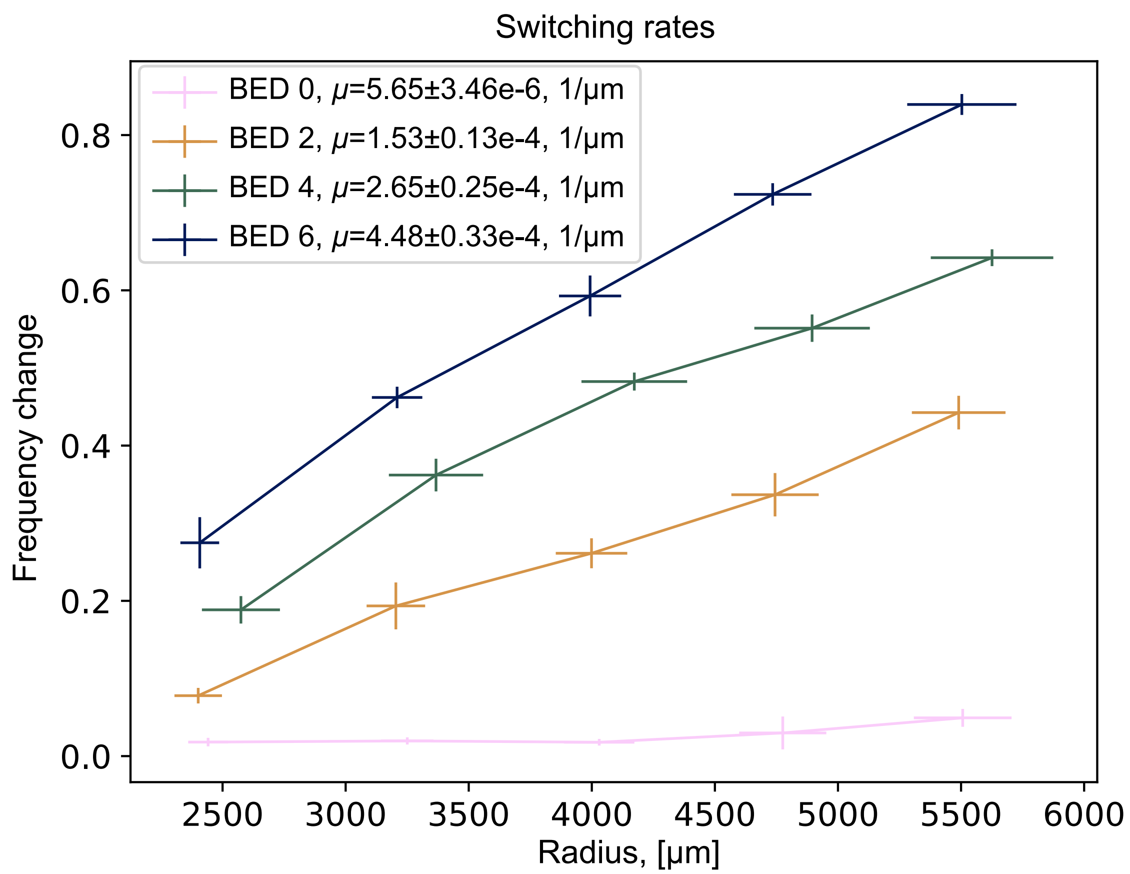

Supplementary Figure 14: **Quantification of mutation rates.** Frequency change over colony radius fitted with rate equations (see Methods section) to get mutation rates for different concentrations of β-Estradiol (mean $\pm$ SD).$n = 6$ independent colonies for each condition.

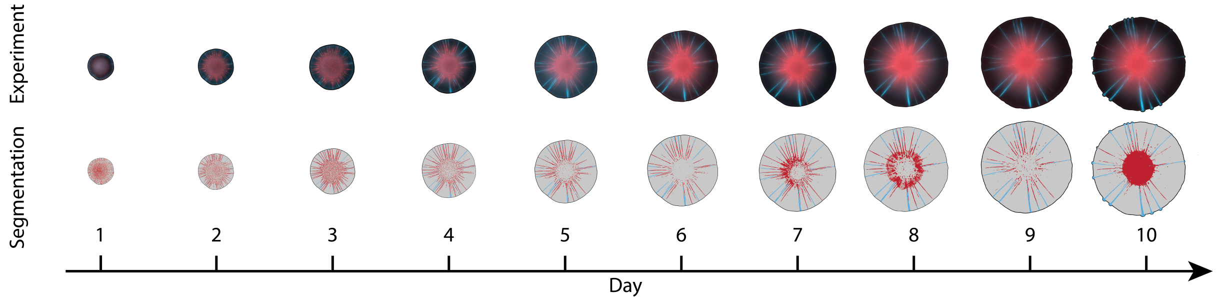

Supplementary Figure 15: **Colony time lapse.** Time lapse images of one colony, and their segmentations. Fitness cost $s = 0.013 \pm 0.006$, mutation rate - $\mu = 2.65 \pm 0.25 \times 10^{-4} \, \frac{1}{\mu\text{m}}$.

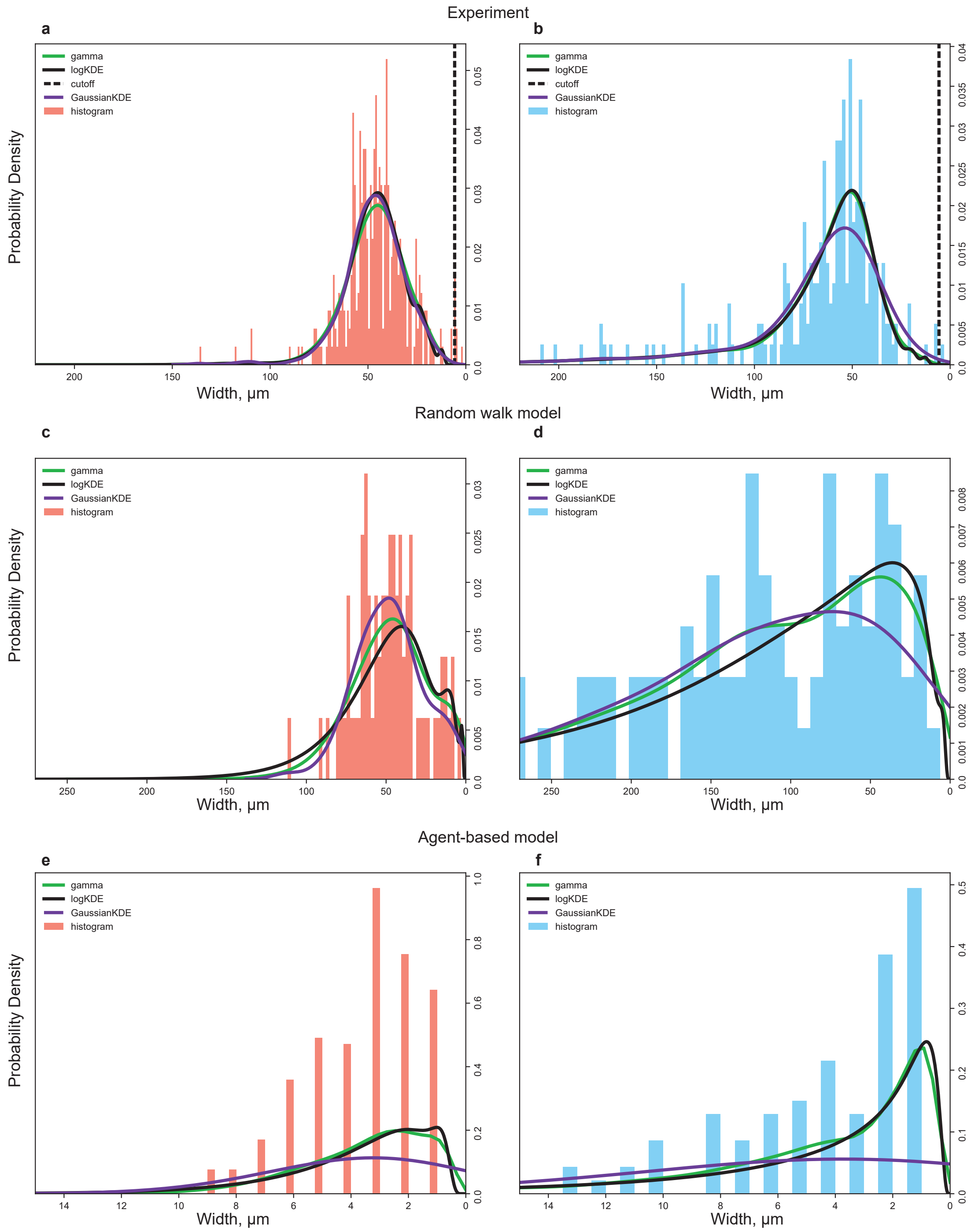

Supplementary Figure 16: **Clone width distribution**. Representative uncompensated (left) and compensated (right) clone width distribution for the experimental data (**a** and **b**) (Figure 2), random walk model (**c** and **d**) (Figure 5) and agent-based simulation (**e** and **f**) (Figure 6). Bars show the histogram of the data. Probability density distribution estimation using gamma function kernel [2] - red line, conventional Gaussian kernel - blue line, and log-transformed Gaussian kernel [3] - solid black line. Dashed lines in the panels **a** and **b** show the single-pixel value cut-off applied on experimental data to avoid segmentation-associated artifacts.

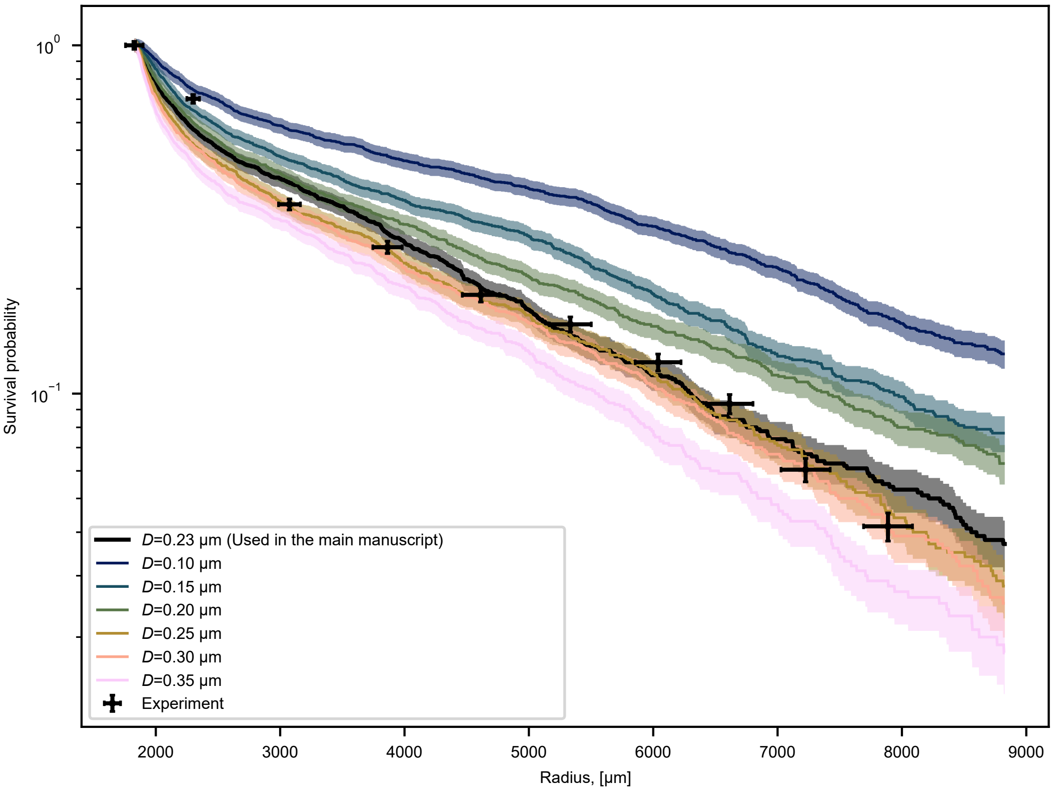

Supplementary Figure 17: **Simulations with different diffusion coefficients.** Survival probability comparison of random walk simulations with different diffusion coefficients, representing different strength of genetic drift. n = 10000 simulated clones for each condition. Experimental data is identical to Figure 2f, n = 18 independent colonies. Shaded areas indicate Poisson distribution SD.

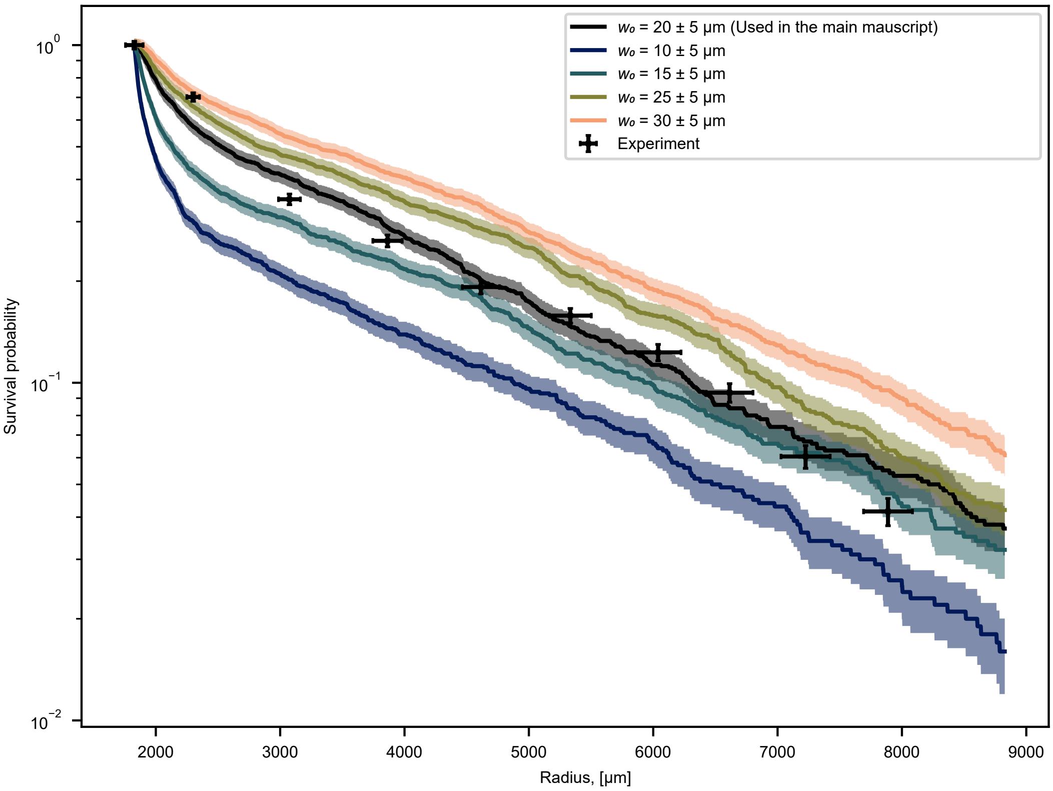

Supplementary Figure 18: **Simulations with different initial clone width.** Survival probability comparison of random walk simulations with different initial clone width. $n = 10000$ simulated clones for each condition. Widths are normally distributed with mean and variance specified in the legends. Experimental data is identical to Figure 2f, $n = 18$ independent colonies. Shaded areas indicate Poisson distribution SD.

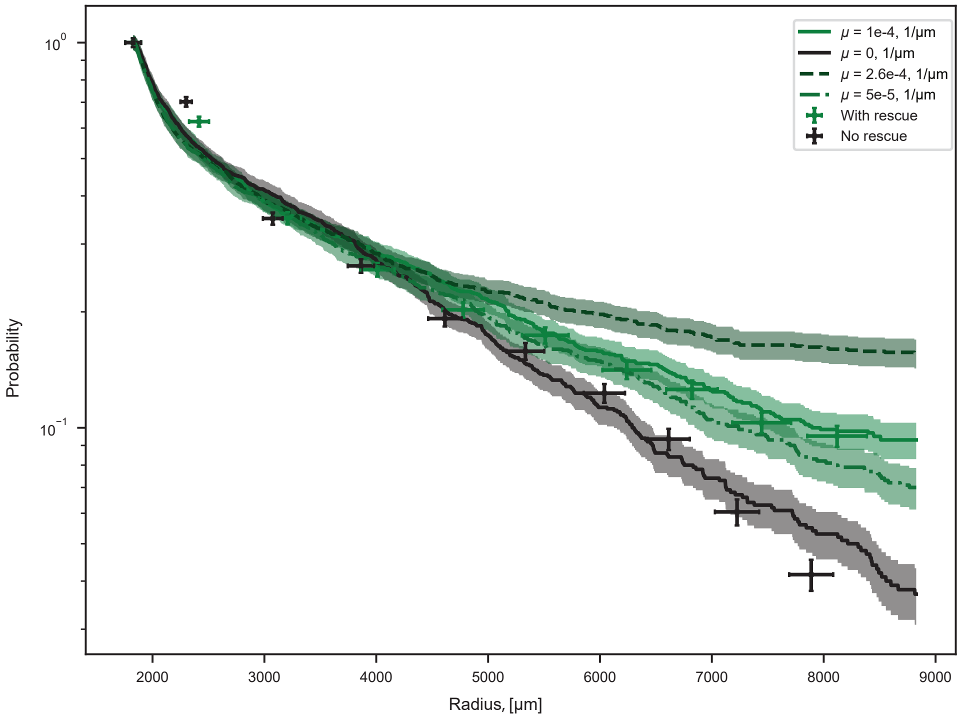

Supplementary Figure 19: **Comparison of simulations with different mutation rates**. Survival probability of clones. Points represent experimental data (mean ± SD, identical to Figure 2f, $n = 18$ independent colonies), solid lines - simulation with parameters presented in the main manuscript. Other line styles correspond to simulations with different mutation rates. $n = 10000$ simulated clones for each condition. Shaded area indicate Poisson distribution SD.

Supplementary References

[1] Korolev, K. S. *et al.* Selective sweeps in growing microbial colonies. *Physical Biology* **9**, 026008 (2012).

[2] Hoffmann, T. & Jones, N. S. Unified treatment of the asymptotics of asymmetric kernel density estimators (2015).

[3] Jones, A. T., Nguyen, H. D. & McLachlan, G. J. logKDE: log-transformed kernel density estimation. *Journal of Open Source Software* **3**, 870 (2018).
