# Supplementary material for: Evolutionary rescue of resistant mutants is governed by a balance between radial expansion and selection in compact populations
Source: Nat Commun. 2022 Dec 23;13:7916. doi: 10.1038/s41467-022-35484-y (PMC9789051; doi:10.1038/s41467-022-35484-y)
Supplement: Supplementary file 3 — Description of Additional Supplementary Files [file 41467_2022_35484_MOESM3_ESM.pdf]

Description of Additional Supplementary Files

Supplementary Movie 1.
Single-cell compensatory mutation of yJK26.
Single-cell resolution time lapse of yJK26 yeast cell undergoing compensatory mutation. Red yJK26 cell (resistant uncompensated) in the bottom undergoes a “synthetic mutation” changing its colour to cyan (resistant compensated). The mutation is inherited by its offspring, so its daughter cell is also cyan-fluorescent.

Supplementary Movie 2.
Agent-based simulation with a triggered mutation at a specific radius.
Agent-based simulation of tumour radial expansion. Simulation initiated similar to the inoculum of the main experiment with a hollow dense ring of cells, with sufficiently spaced single slower-growing mutants (red) interspersed at the periphery. Resistant to sensitive cell fraction is 0.166. Red cells have a fitness cost of $s = 0.21$. Each red clone deterministically gets a single-cell compensatory mutation offsetting the fitness cost and changing cell colour to blue.

Supplementary Movie 3.
Agent-based simulation with randomly occurring compensatory mutations.
Agent-based simulation of tumour radial expansion. Simulation initiated similar to the inoculum of the main experiment with a hollow dense ring of cells, with sufficiently spaced single slower-growing mutants (red) interspersed at the periphery. Resistant to sensitive cell fraction is 0.166. Red cells have a fitness cost of $s = 0.21$. Red cells can stochastically mutate changing their colour to blue and compensating for the fitness cost.
