# Supplementary material for: Evolutionary rescue of resistant mutants is governed by a balance between radial expansion and selection in compact populations
Source: Nat Commun. 2022 Dec 23;13:7916. doi: 10.1038/s41467-022-35484-y (PMC9789051; doi:10.1038/s41467-022-35484-y)
Supplement: Supplementary file 7 — Reporting Summary [file 41467_2022_35484_MOESM7_ESM.pdf]

nature portfolio

Corresponding author(s): Jona Kayser and Oskar Hallatschek

Last updated by author(s): Nov 28, 2022

Reporting Summary

Nature Portfolio wishes to improve the reproducibility of the work that we publish. This form provides structure for consistency and transparency in reporting. For further information on Nature Portfolio policies, see our Editorial Policies and the Editorial Policy Checklist.

Statistics

For all statistical analyses, confirm that the following items are present in the figure legend, table legend, main text, or Methods section.

n/a	Confirmed	
☐	☒	The exact sample size (*n*) for each experimental group/condition, given as a discrete number and unit of measurement
☐	☒	A statement on whether measurements were taken from distinct samples or whether the same sample was measured repeatedly
☐	☒	The statistical test(s) used AND whether they are one- or two-sided<br>*Only common tests should be described solely by name; describe more complex techniques in the Methods section.*
☒	☐	A description of all covariates tested
☒	☐	A description of any assumptions or corrections, such as tests of normality and adjustment for multiple comparisons
☐	☒	A full description of the statistical parameters including central tendency (e.g. means) or other basic estimates (e.g. regression coefficient) AND variation (e.g. standard deviation) or associated estimates of uncertainty (e.g. confidence intervals)
☐	☒	For null hypothesis testing, the test statistic (e.g. *F*, *t*, *r*) with confidence intervals, effect sizes, degrees of freedom and *P* value noted<br>*Give P values as exact values whenever suitable.*
☒	☐	For Bayesian analysis, information on the choice of priors and Markov chain Monte Carlo settings
☒	☐	For hierarchical and complex designs, identification of the appropriate level for tests and full reporting of outcomes
☒	☐	Estimates of effect sizes (e.g. Cohen's *d*, Pearson's *r*), indicating how they were calculated

Our web collection on statistics for biologists contains articles on many of the points above.

Software and code

Policy information about availability of computer code

Data collection	Microscopy images were collected using Zeis ZEN 3.0 (blue edition), agent-based simulation data produced with PhysiCell V 1.8.0 with BioFVM (V 1.1.6).
Data analysis	Microscopy images were segmented using Ilastik V 1.3.3, data was analyzed with custom algorithms in MATLAB (R2020a) and python 3.8. Image processing was performed using FIJI 2.0.0. All custom code is available on GitLab: https://gitlab.gwdg.de/kayser-lab/aif_isb

For manuscripts utilizing custom algorithms or software that are central to the research but not yet described in published literature, software must be made available to editors and reviewers. We strongly encourage code deposition in a community repository (e.g. GitHub). See the Nature Portfolio guidelines for submitting code & software for further information.

Data

Policy information about availability of data

All manuscripts must include a data availability statement. This statement should provide the following information, where applicable:

- Accession codes, unique identifiers, or web links for publicly available datasets
- A description of any restrictions on data availability
- For clinical datasets or third party data, please ensure that the statement adheres to our policy

Imaging data used in this study is available at https://figshare.com/projects/Aif2022_NatComms/146175.

Human research participants

Policy information about studies involving human research participants and Sex and Gender in Research.

Reporting on sex and gender	No human subjects were involved in this study.
Population characteristics	No human subjects were involved in this study.
Recruitment	No human subjects were involved in this study.
Ethics oversight	No human subjects were involved in this study.

Note that full information on the approval of the study protocol must also be provided in the manuscript.

Field-specific reporting

Please select the one below that is the best fit for your research. If you are not sure, read the appropriate sections before making your selection.

☒ Life sciences ☐ Behavioural & social sciences ☐ Ecological, evolutionary & environmental sciences

For a reference copy of the document with all sections, see nature.com/documents/nr-reporting-summary-flat.pdf

Life sciences study design

All studies must disclose on these points even when the disclosure is negative.

Sample size	The initial number of clones was estimated by manually counting clones from the single-cell resolution images for 12 individual colonies. This is the maximum amount that can be achieved in our experimental setup within a suitable imaging time. The subsequent analysis showed that the given sample size was indeed sufficient.
Data exclusions	Colonies that showed signs of contamination were excluded from the analysis.
Replication	We conducted competitions assays in replicate on a total of 92 colonies in individual wells on 6-well plates, with at least 5 colonies in the same chemical environment. Exact numbers of colonies for each specific chemical environment are presented in the Methods section. All replication attempts were successful.
Randomization	This was not relevant in this work since randomization can have no effect on observables.
Blinding	Blinding was not relevant in this work since no group allocation was performed.

Reporting for specific materials, systems and methods

We require information from authors about some types of materials, experimental systems and methods used in many studies. Here, indicate whether each material, system or method listed is relevant to your study. If you are not sure if a list item applies to your research, read the appropriate section before selecting a response.

Materials & experimental systems

n/a	Involved in the study	
☒	☐	Antibodies
☐	☒	Eukaryotic cell lines
☒	☐	Palaeontology and archaeology
☒	☐	Animals and other organisms
☒	☐	Clinical data
☒	☐	Dual use research of concern

Methods

n/a	Involved in the study	
☒	☐	ChIP-seq
☒	☐	Flow cytometry
☒	☐	MRI-based neuroimaging

Eukaryotic cell lines

Policy information about cell lines and Sex and Gender in Research

Cell line source(s)	Eucaryotes in this study are S. cerevisiae yeast cells based on the W303 laboratory strain.

Authentication	PCR and Sanger sequencing
Mycoplasma contamination	Not applicable to yeast
Commonly misidentified lines (See ICLAC register)	Not applicable to yeast
